# SARS-CoV-2 adsorption on suspended solids along a sewerage network: mathematical model formulation, sensitivity analysis, and parametric study

**DOI:** 10.1007/s11356-021-16528-0

**Published:** 2021-09-17

**Authors:** Margaritis Kostoglou, Maria Petala, Thodoris Karapantsios, Chrysostomos Dovas, Emmanuel Roilides, Simeon Metallidis, Anna Papa, Efstratios Stylianidis, Agis Papadopoulos, Nikolaos Papaioannou

**Affiliations:** 1 Laboratory of Chemical and Environmental Technology, Department of Chemistry, Aristotle University of Thessaloniki, 54124 Thessaloniki, Greece; 2 Laboratory of Environmental Engineering & Planning, Department of Civil Engineering, Aristotle University of Thessaloniki, 54 124 Thessaloniki, Greece; 3 Faculty of Veterinary Medicine, School of Health Sciences, Aristotle University of Thessaloniki, 54124 Thessaloniki, Greece; 4 Infectious Diseases Unit and 3rd Department of Pediatrics, Aristotle University School of Health Sciences, Hippokration Hospital, 54642 Thessaloniki, Greece; 5 Department of Haematology, First Department of Internal Medicine, Faculty of Medicine, AHEPA General Hospital, Aristotle University of Thessaloniki, 54636 Thessaloniki, Greece; 6 Department of Microbiology, Medical School, Aristotle University of Thessaloniki, 54124 Thessaloniki, Greece; 7 School of Spatial Planning and Development, Faculty of Engineering, Aristotle University of Thessaloniki, 54124 Thessaloniki, Greece; 8 EYATH S.A., Thessaloniki Water Supply and Sewerage Company S.A., 54636 Thessaloniki, Greece

**Keywords:** SARS-CoV-2, Wastewater epidemiology, Modeling, adsorption, Virus concentration rationalization, Sewerage network

## Abstract

Accounting for SARS-CoV-2 adsorption on solids suspended in wastewater is a necessary step towards the reliable estimation of virus shedding rate in a sewerage system, based on measurements performed at a terminal collection station, i.e., at the entrance of a wastewater treatment plant. This concept is extended herein to include several measurement stations across a city to enable the estimation of spatial distribution of virus shedding rate. This study presents a pioneer general model describing the most relevant physicochemical phenomena with a special effort to reduce the complicated algebra. This is performed both in the topology regime, introducing a discrete-continuous approach, and in the domain of independent variables, introducing a monodisperse moment method to reduce the dimensionality of the resulting population balance equations. The resulting simplified model consists of a large system of ordinary differential equations. A sensitivity analysis is performed with respect to some key parameters for a single pipe topology. Specific numerical techniques are employed for the integration of the model. Finally, a parametric case study for an indicative—yet realistic—sewerage piping system is performed to show how the model is applied to SARS-CoV-2 adsorption on wastewater solids in the presence of other competing species. This is the first model of this kind appearing in scientific literature and a first step towards setting up an inverse problem to assess the spatial distribution of virus shedding rate based on its concentration in wastewater.

## Introduction

The majority of wastewater-based epidemiology (WBE) studies aiming at population-wide surveillance for SARS-CoV-2 focuses on the detection of viral genetic remnants in the sewage entering a wastewater treatment plant (la Rosa et al. 2020; Orive et al. 2020; Medema et al. 2020; Ahmed et al. 2020; Nemudryi et al. 2020; Rimoldi et al. 2020; Corpuz et al. 2020; D’Aoust et al. 2021; Saguti et al. 2021). Such virus signals have been found to indicate global community infections but, apparently, they are unable to identify local spots of rising infections within the community. Even for global estimations, the situation gets complicated by the survival and partitioning (between liquid and solid phase) properties of viruses in the aqueous environments of sewage which can mask the actual virus profile and so mislead their quantification (Corpuz et al. 2020; Li et al. 2021a,b).

Virus survival and partitioning are two different things (Grant et al. 1993). Survival refers to virus preservation as infectious viral particles and specifically on their inactivation or disruption, which render them inert and harmless. Partitioning refers to virus particles (or virus fragments) adsorption onto the surface of porous solids suspended in wastewater, which makes them passive or just inaccessible. Inactivation can be due to biological effects, ageing, thermal effects, or chemical effects. For instance, enveloped viruses such as SARS-CoV-2, because of their lipid bilayer membrane surrounding the virus protein capsid, can be inactivated and disrupted to fragments by detergents and organic solvents present in wastewaters (Ye et al. 2016). Adsorption, on the other hand, of virus particles and fragments can be influenced by a number of transport and physicochemical parameters among which are wastewater flow intensity, solids concentration, pH, ionic strength, and organic load. Organic load refers to chemical organic matter, e.g., hydrocarbons, carbohydrates, amino acids, humic substances, and biological organic matter, e.g., bacteria, protozoa, other viruses, that may also adsorb on suspended solids. There is enough evidence in literature demonstrating that dissolved organic matter competes for the same binding sites with virus particles and fragments but bonded organic matter provides new hydrophobic binding sites, so the overall effect of organic matter is not easy to quantify (Schijven and Majid Hassanizadeh 2000). In general, little is known on the role of environmental conditions on virus persistence in aquatic systems (Waldman et al. 2020). Recently, relevant knowledge was acquired on how environmental parameters affect the fate of biocides in wastewater and potable water distribution systems (because of adsorption, ion exchange, etc.) at conditions met in the International Space Station (Giakisikli et al. 2017; Petala et al. 2017, 2020).

If one cares to detect virus genomic regions in wastewater for epidemiological surveillance reasons, it does not matter if viruses are inactivated or disrupted. Therefore, concerns about virus survival are of no interest. On the contrary, adsorption to solid particles of both whole viruses and virus fragments is of great significance because the degree of adsorption is associated directly with the degree of recovery from wastewater for virus detection and quantification (Hvitved-Jacobsen et al. 2013). It must be mentioned here that determining the extent of recovery (desorption of adsorbed species) by spiking techniques is inaccurate because they are applied to samples that have (most of) their adsorption sites already saturated with adsorbed species. Adsorption and desorption of spiked material would be completely different if the sample was taken fresh at the location of shedding.

It is noted that virus fragments can be also adsorbed to biofilms found attached on the walls of wastewater pipes and this contribution must, in principle, be considered. However, at this stage of model development, virus fragment adsorption by biofilms is not included in the model that focuses on simulating SARS-CoV-2 virus adsorption on wastewater suspended solids. However, the total mass of biofilm remains practically unchanged at the time scale of the residence time of virus parts in the piping system and the adsorption on this mass is always close to equilibrium (saturation). Therefore, the contribution of biofilms on the adsorption dynamics of virus parts cannot be comparable to the effect of the continuously entering the flow system suspended solid particles which act as “unsaturated” adsorbents. In addition, due to their small size and internal porous network suspended solid particles have a much larger surface area than the biofilms. In this respect, at this stage of model development, virus part adsorption by biofilms is considered negligible.

When talking of adsorption, both the effects of attachment and detachment to solids are considered, because such phenomena can be either irreversible or reversible (Schijven and Majid Hassanizadeh 2000). Detachment of the virus can be brought about by changes in the wastewater chemistry, for instance, an increase in pH (Loveland et al. 1996; Mayotte 2016). This can occur when wastewater from a pipe in a sewerage network mixes with different quality wastewater of another pipe in the network, e.g., coming from a different city region.

In many cities, topographic and terrain-driven peculiarities generate the need for operating combined gravity-driven and pressure-driven sewerage networks. Although gravity-driven networks are simpler to construct, cheaper to implement, and have lower operational costs, pressure-driven networks offer the advantage of tight pipelines that eliminate odors of pumped wastewater and, more importantly, shorten the sewage retention time in pipelines (Huskie 2000). Infiltration and exfiltration are particularly difficult to determine, given the fact that in cities with a population of more than 500,000, the pipeline’s length of pressure installations extends to several tenths of kilometers. Assessing the infiltration/exfiltration by means of inspections is cost- and time-intensive and can provide only snapshots of the conditions prevailing, despite the advances in tracer methods and CCTV (Tscheikner-Gratl et al. 2019). Hence, modelling, be it statistical, heuristic, or machine learning, provides a feasible option, despite its complexity. Still, for installations in larger cities, the mathematical formulation of such lengthy installations with varying geometric, transport and physicochemical conditions requires detailed 3D spatial models that lead to unacceptably long computation times. For this reason, simpler models have been suggested as convenient alternatives that sacrifice some geometrical detail at the benefit of much less computation time while maintaining satisfactory calculation accuracy (Siwicki et al. 2020).

As of April 2020, our interdisciplinary team in the Aristotle University of Thessaloniki (Greece) in cooperation with Thessaloniki’s Water Supply and Sewerage company quantifies SARS-CoV-2 in sewage right before they enter the main wastewater treatment plant of the city. On this account, a comprehensive mathematical model was developed for the adsorption of virus parts onto porous solids suspended in wastewater along a city sewerage network (Petala et al. 2021). That work was meant to set up the general framework of the model with emphasis (i) on the large-scale topological complexity of actual sewage networks and (ii) on elaborating physicochemical phenomena in adsorption to eliminate possible uncertainties in virus quantification.

Efforts are currently focused on a more detailed spatial analysis of the SARS-CoV-2 load in different parts of Thessaloniki. In the words of CDC, “Using sampling points upstream from wastewater treatment plants to monitor sub-sewer shed infection trends requires additional work to understand the boundaries and unique characteristics of that area before it can be used for wastewater surveillance.” (CDC 2021). The present work is part of these efforts and refers to extending and refining the previous model (Petala et al. 2021) to include additional transport and physicochemical parameters of significance and also realistic small-scale topological details of sewerage networks. The formulation of the mathematical model is followed by a sensitivity analysis to display the significance of individual key parameters in the response of the system. Finally, a parametric study is presented for an indicative set of parameters showing important features of virus load evolution along the examined sewerage network, demonstrating the capacity and purposefulness of the model. Although this work is oriented towards SARS-CoV-2, the proposed framework can have broader use in WBE in relation to other viruses, chemicals, drugs, etc. To the best of our knowledge, this is the first time that WBE is addressed through a fine-grain resolution mathematical model with respect to discrete topological features within sewerage networks.

## Methods

### Problem formulation

The model consists of two modules: the first is a physicochemical module that describes the applicable physicochemical (molecular) and transport phenomena whereas the second is a topological module that describes the piping network topology.

### Upgrade of physicochemical model

The previous model (Petala et al. 2021) handles the suspended solid particles as a single bulk phase with uniform properties without considering their size. However, at the time scale of interest for wastewater flow in a sewage piping network (from hours to days), there may be a distribution of adsorbate in porous particles which depends on particle size. For this reason, in the present work, the particles entering the flow are considered discrete entities of different size which can be characterized (in a mean-field approach) by a number density function g(m) where m is the particle mass and g(m)dm is the mass of particles entering the flow per unit time having a mass between m and m+dm. The suspended solid mass entering rate is denoted as ${M}_{in}={\int}_0^{\infty }g(m) dm$. Let us denote as C the bulk concentration of virus parts or fragments (VP) and q the adsorbed quantity of VP per unit mass of solids. Under equilibrium conditions, the physical forces (electrostatic and Van der Waals forces) between virus parts and porous solid walls lead to an excess of adsorbed concentration over bulk concentration of VP. This is quantitatively described through the so-called adsorption isotherm function which has the form *q*_e_=*I*(*C*_e_), where *q*_e_ is the adsorbed quantity at equilibrium (Ruthven 2004). The driving force for adsorption is the instantaneous difference between the actual value of q and the equilibrium value *q*_e_. In order to eliminate this difference, the VP are transferred from the bulk liquid to the external particle surface through convective diffusion and from the external particle surface to the particle interior through pore and surface diffusion (Loukidou et al. 2004; Karapantsios et al. 2005). Pore diffusion is a liquid phase process, and the corresponding diffusivity can be theoretically found by a combination of bulk solute diffusivity and adsorption isotherm function. Surface diffusion refers to the adsorbed material onto pores surface and there is no theory for predicting the corresponding diffusivity. The computation of adsorption dynamics by a spherical particle requires the solution of a complicated partial differential equation. A detailed solution method and data interpretation for a well-characterized physical system can be found in the study performed by Kyzas et al. (2010). The mathematical problem can be greatly simplified by applying the so-called Linear Driving Force approach (Tien 1994). Roughly speaking, a presumed concentration profile of q is employed, and mathematical analysis leads to the following ordinary differential equation for the evolution of *q* (for a spherical particle):

1 $$ \frac{dq}{dt}=\frac{15}{\frac{5r}{h}+\frac{r^2}{D_{eff}}}\left(I(C)-q\right) $$

where *t* is the time, *r* is the radius of the spherical particle, *D*_eff_ is the effective diffusivity of the VP in the particle, and *h* is the mass transfer coefficient from the bulk liquid to the external particle surface. The constants 5 and 15 in Eq. (1) are the outcomes of the application of the linear driving force approach. The effective diffusivity contains both pore and surface diffusion contributions and the existence of a functional dependence form of *D*_eff_(*q*) has been well recognized in the literature (Ruthven 2004). The above functional form can be generalized by adding a particle mass dependence for spatially non-uniform particles (e.g., a large particle with a core region inaccessible by VP).

Particles acquire a relative motion within the liquid due to gravitational force and to turbulent inertia (inability of the particle to follow the high frequency turbulent velocity fluctuations of the liquid). An estimation of the root mean square, *u*_t_, of the turbulent induced relative particle-liquid velocity is given by the following relation (Friedlander 1957):

2 $$ {u}_t=\sqrt{2}\left|1-{\rho}_l/{\rho}_p\right|\frac{I_TV}{\lambda_K\gamma } $$

where *ρ*_1_ and *ρ*_p_ are the liquid and particle density, respectively, *I*_T_ is the turbulence intensity, *λ*_K_ is the Kolmogorov turbulence microscale, V the average cross-section liquid velocity, and γ is the ratio of friction factor to the mass of the particles. The sedimentation particle velocity (using Stokes law corrected by Oseen (Happel and Brenner 1965) using the correction factor 1+3N_Re_/8 where *N*_Re_ is the particle Reynolds number based on Stokes velocity) must be added to *u*_t_ to find the total particle-liquid relative velocity *u*. All the above quantities can be computed given the pipe radius, the flow rate, and the particle mass. The mass transfer coefficient, *h*, can be found using the relation for convective transfer (Sherwood et al. 1977):

3 $$ {N}_{Sh}=2{\left(1+0.325{N}_{Pe}^{2/3}\right)}^{1/2} $$

where *N*_sh_ is the Sherwood number, and *N*_*P*e_ is the mass transfer Peclet number computed for velocity *u*. Therefore, the model takes the total particle-liquid relative velocity implicitly into account through the Peclet number. The equation holds for particle Reynolds number, based on the above referred relative velocity, smaller than one.

Summarizing, in the most general consideration

4 $$ \frac{dq}{dt}=\frac{15}{\frac{5r}{h(m)}+\frac{r^2}{D_{eff}\left(q,m\right)}}\left(I(C)-q\right)=G\left(q,C,m\right) $$

The particle radius *r* can be computed by the particle mass and density. In the case of a non-spherical but nearly isotropic particle, the equivalent radius can be considered *r*. There are only empirical relations for the dependence of *D*_eff_ on q (a usual relation is *D*_eff_=π_1_(1+π_2_q)^-π3^ where π_1_, π_2_, and π_3_ are empirical parameters (Ruthven 2004). It is worth noting that the present model can predict even the desorption of adsorbed VP depending on the local conditions along the flow path.

### Upgrade of topological model

The sewage piping topology is an essential aspect of the model. In our previous work, the two limits of the fully discrete model on one hand, which accurately reconstructs the entire city piping network and of the continuous model on the other hand, which focuses on the flow path along a main pipe, are formulated. In this new approach, we develop a generalization creating a whole hierarchy of models between the two limits. The piping system between the smallest pipe (discrete model) and the largest one (main flow path) is classified in levels. Level 1 corresponds to large size conduits connecting regional piping networks to the main conduit (largest conduit of all). Level 2 corresponds to smaller conduits connected to regional conduits of level 1. The position of the model in the hierarchy is denoted by a single number *N*_L_ denoting the higher piping level, which is handled in detail by the model.

**Fig. 1:**
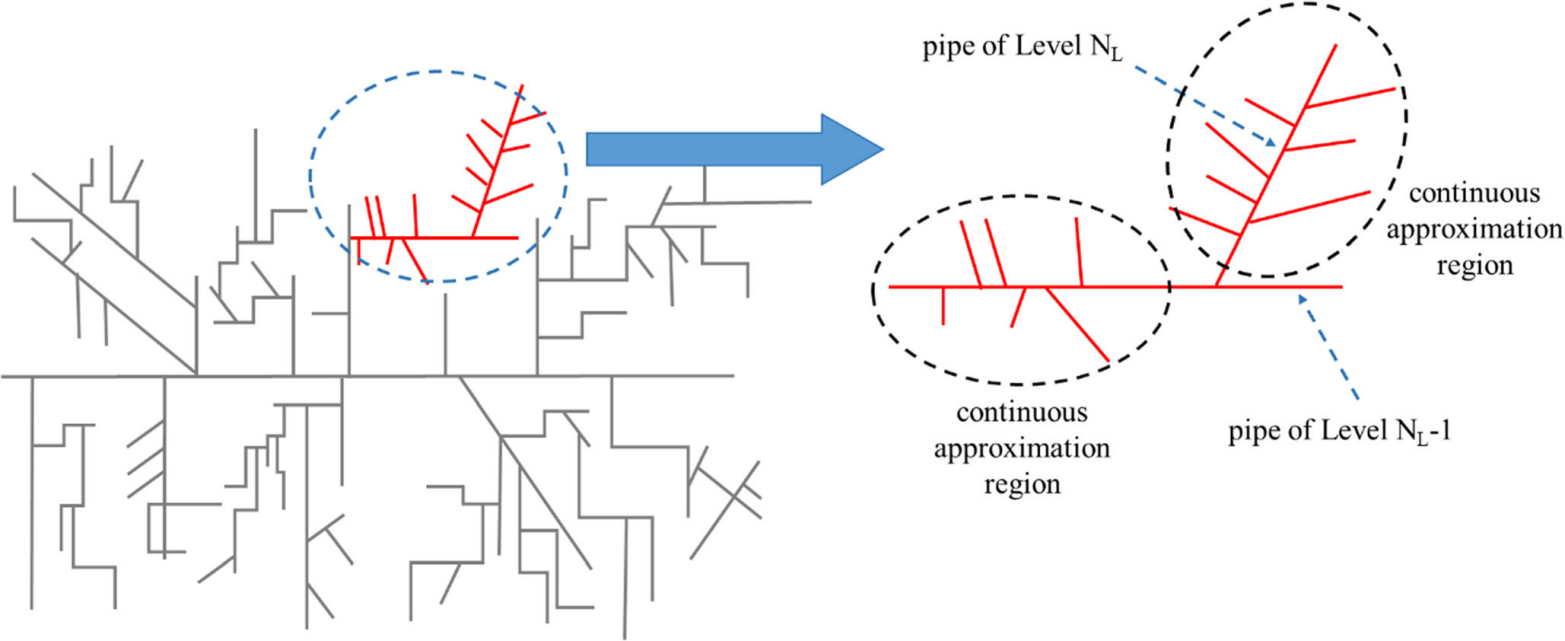
Description of the topology structure of a sewerage piping system focusing on the connection between a level *N*_*L*-1_ and a level *N*_L_ pipes

A schematic of the connection between a level *N*_L_ and a level *N*_L_-1 pipe is shown in Fig. 1 in order to facilitate topological model understanding. The two limiting models presented in our previous work (Petala et al. 2021) are characterized by *N*_L_=*N*_max_ (being the highest-level number of the piping system) for the discrete model and *N*_L_=0 for the fully continuous main flow path model.

A model of index *N*_L_ denotes that pipes of level up to *N*_L_ are explicitly handled. The connections between pipes of two consecutive levels up to *N*_L_ are handled in a discrete manner whereas the connections between levels *N*_L_ and *N*_L+1_ and the connections between pipes of non-consecutive levels are treated using the continuous approach. This procedure (depending on the connectivity of the piping system) leads to a discrete-continuous or discrete formulation for the pipes of levels 0 to *N*_L-1_ and continuous formulation for the level *N*_L_. It is noted that there are commercial fluid dynamics codes to simulate the detailed piping system. The present physicochemical model can be incorporated into such codes. Yet, here the scope is not to develop such a sophisticated code but a reduced model capable of exploring experimental measurements. An important contribution of this work is the proposed hierarchical (discrete-continuous) approach to describe the piping network. The only flow detail used herein is the one of turbulent flow which is the case for all main wastewater pipes in practice irrespective of the origin of flow generation.

### Model development

The model is a pseudo-steady state one, despite the temporal variation of input variables (flow rates, concentration, etc.). The validity condition of this approach is to have no significant temporal variation at the time scale of fluid residence time in the piping network. This may set a limitation in cases of short-duration rains in cities with combined sewer systems but it satisfies most other conditions pretty well. In other terms, it can be assumed that the model produces averages of the quantities over a time equal to the total residence time of the fluids in the system. The transformation of the discrete connections to continuous ones is made by replacing a sum of weighted Dirac functions by a single continuous function. This is valid in the limit of small variation of the problem state variables between two consecutive connections. This is rather common inasmuch as consecutive connections refer to nearby regions. In order to better demonstrate the new hierarchical topological approach, the governing equations are presented for a topological model of *N*_L_=1 with no connectivity between the pipe at level 0 and the pipes at levels above 1. This suggests a discrete formulation for the level 0 flow path and continuous formulation for the flow paths of level 1 (let us denote as *N*_*1*_ the number of these flow paths).

#### *Equations for pipes of level 1*

Let *z* be the spatial coordinate along each pipe, *L* is the pipe length, *V(z)* is the average liquid velocity, and *S(z)* is the (non-uniform in general) flow cross-section. The rate of liquid mass, suspended solids mass distribution, and rate of VP mass entering the pipe per unit length at position z is described by the functions *F*_in_(*z*), *g*_in_(*m,z*), and *P*_*in*_(*z*). The moments of function *g*_in_(*m*) are defined as ${K}_{in,i}={\int}_0^{\infty }{m}^i{g}_{in}(m) dm$. It is important to notice that each solid particle is characterized by its mass and by the load of adsorbed VP. The total particle population is characterized by the bivariate particle mass distribution *f(m,q)* which denotes the mass concentration of solid particles having mass between m and *m+dm* and VP load between *q+dq*. It must be noted that *q* does not refer to the total amount of adsorbed VP on a particle but it is defined per unit mass of the particle. An additional subscript j is added to the above variables in order to designate to which one of the level 1 pipes they refer to (*j*=1 to *N*_1_). The governing equations are the following:

Liquid balance

5 $$ \frac{d{S}_j{V}_j}{d{z}_j}={F}_{in,j}\left({z}_j\right) $$

VP balance

6 $$ \frac{d{S}_j{V}_j{C}_j}{d{z}_j}={P}_{in,j}(z)-{S}_j{\int}_0^{\infty }{\int}_0^{\infty }G\left(q,{C}_j,m\right){f}_j\left(q,m\right) dqdm $$

Solid particles balance

7 $$ \frac{\partial {S}_j{V}_j{f}_j\left(m,q,z\right)}{\partial {z}_j}+{S}_j\frac{\partial G\left(q,C,m\right){f}_j\left(m,q,z\right)}{\partial q}={g}_{in,j}\left(m,z\right)\delta (q) $$

where δ is the Dirac delta function that accounts for the fact that solids enter the paths with no adsorbed virus parts on them, and *C* is the liquid phase VP concentration. This assumption can be relaxed, however, in cases that more specific information about this quantity is available. The population balance approach adopted here has the advantage that additional particle level phenomena, such as aggregation and breakage, can be readily incorporated into the model (Ramkrishna 2000).

#### Equations for main flow path of level 0

The main flow path has a number of *N*_1_ connections to the level 1 pipes. The governing equations between these connections (subscript “*F*” denotes the main flow path) are as follows.

Liquid balance

8 $$ \frac{d{S}_F{V}_F}{dz}=0 $$

VP balance

9 $$ \frac{d{S}_F{V}_F{C}_F}{dz}=-{S}_F{\int}_0^{\infty }{\int}_0^{\infty }G\left(q,{C}_F,m\right){f}_j\left(q,m\right) dqdm $$

Suspended solid particles balance

10 $$ \frac{\partial {S}_F{V}_F{f}_F\left(m,q,z\right)}{\partial z}+{S}_F\frac{\partial G\left(q,{C}_F,m\right){f}_F\left(m,q,z\right)}{\partial q}=0 $$

In addition. the following balances hold at each of the *N*_1_ connections:

11a $$ \varDelta \left({S}_F{V}_F\right)={\left({S}_j{V}_j\right)}_{z_j={L}_j} $$

11b $$ \varDelta \left({S}_F{V}_F{C}_F\right)={\left({S}_j{V}_j{C}_j\right)}_{z_j={L}_j} $$

11c $$ \varDelta \left({S}_F{V}_F{f}_F\left(m,q\right)\right)={\left({S}_j{V}_j{f}_j\left(m,q\right)\right)}_{z_j={L}_j} $$

where the operator Δ denotes the difference in main flow path variables before and after the connection.

### Order reduction of the mathematical problem

The above-formulated problem consists of a system of several bivariate population balances (partial differential equations) that has no analytical solution. The typical approach to the numerical solution is the employ finite difference equations. Considering the hyperbolic type of the problem specially designed discretization techniques are needed (Kostoglou and Karabelas 2004). In any case, a dense discretization in the *q* direction and a coarse one in the *m* direction are necessary. This leads to a system of thousands of ordinary differential equations after discretization of the complete model. It is clear that such a computational burden is incompatible with the scope of the present model and with the uncertainty of the parameter values. The order reduction of the mathematical problem is the only reasonable way to proceed. This approach retains the complete parametric dependence of the problem but reduces the required computational effort by sacrificing only output details such as the complete particle mass and VP load distribution.

Let us denote as follows the lowest order moments of the bivariate particle distribution

12a $$ {M}_{-1,0}={\int}_0^{\infty }{\int}_0^{\infty}\frac{1}{m}f\left(m,q\right) dqdm $$

12b $$ {M}_{0,0}={\int}_0^{\infty }{\int}_0^{\infty }f\left(m,q\right) dqdm $$

12c $$ {M}_{0,1}={\int}_0^{\infty }{\int}_0^{\infty } qf\left(m,q\right) dqdm $$

The variable *M*_-1,0_ is the total number concentrations of the solids, the variable *M*_0,0_ is their total mass concentration, and the variable M_0,1_ is the concentration of adsorbed VP. The ratio *q*_ave_=*M*_0,1_/*M*_0,0_ is the average value of q among all particles and the ratio *m*_ave_*=M*_*0*,0_*/M*_-1,0_ is the average particle mass.

Applying the above integral operators to equation (7) using the following integration by part rule (for *i*=0 and 1)

13 $$ {\int}_0^{\infty }{x}^i\frac{\partial Y(x)}{\partial x} dx={\int}_0^{\infty }{x}^i dY(x)={\left[{x}^iY(x)\right]}_{x=0}^{x=\infty }-i{\int}_0^{\infty }Y(x){x}^{i-1} dx=-i{\int}_0^{\infty }Y(x){x}^{i-1} dx $$

leads to

14a $$ \frac{d{S}_j{V}_j{M}_{-1,0,j}\left({z}_j\right)}{d{z}_j}={K}_{in,-1,j}\left({z}_j\right) $$

14b $$ \frac{d{S}_j{V}_j{M}_{0,0,j}\left({z}_j\right)}{d{z}_j}={K}_{in,0,j}\left({z}_j\right) $$

14c $$ \frac{d{S}_j{V}_j{M}_{0,1,j}\left({z}_j\right)}{d{z}_j}={S}_j{\int}_0^{\infty }{\int}_0^{\infty }G\left(q,{C}_j,m\right){f}_j\left(m,q,{z}_j\right) dqdm $$

The resulting system of ordinary differential equations has no closure because of the existence of double integrals over the bivariate particle distribution function. In order to overcome this problem, a shape for f must be chosen. Among the several available choices (i.e., lognormal, Gamma, discrete size distribution), the simplest one is the so-called monodisperse approach (Kostoglou and Karabelas 2004). According to this approach, the following expression is assigned to particle distribution *f(m,q)=M*_0,0_δ(*m-m*_ave_)*δ*(*q-q*_ave_). Substitution in the integral in Eq. (14c) yields:

15 $$ \frac{d{S}_j{V}_j{M}_{0,1,j}\left({z}_j\right)}{d{z}_j}={S}_jG\left({q}_{ave},{C}_j,{m}_{ave}\right){M}_{0,0}\left({z}_j\right) $$

Then Eq. (6) takes the form

16 $$ \frac{d{S}_j{V}_j{C}_j}{d{z}_j}={P}_{in,j}\left({z}_j\right)-{S}_jG\left({q}_{ave},{C}_j,{m}_{ave}\right){M}_{0,0}\left({z}_j\right) $$

Combined with equations *q*_ave_=*M*_0,1_*/M*_0,0_ and *m*_ave_=*M*_*0*,0_*/M*_-1,0_, the system of 3N_1_+3 ordinary differential equations are closed and can be solved numerically in the z direction for the evolution of the average particle properties along with the flow. It is important to notice that the method is not based on an a priori assumption of monodispersity. Actually, complete size distribution is considered but results are produced only for its (approximate) average values.

### Evolution of other factors along the flow path

In our earlier work (Bitton 1975; Petala et al. 2021), physicochemical parameters were considered only as factors affecting the adsorption equilibrium of virus parts. However, it is known that adsorption equilibrium and kinetics of all molecular species may be affected by physicochemical parameters either in the bulk liquid (e.g., pH, temperature, and ionic activity) or in the pores of the suspended solids (e.g., competing or synergistic adsorption) (Bitton 1975). In the case of more complicated adsorbates (e.g., viruses which are actually colloidal particles), contradictory information is found in the literature regarding the influence of organic matter (Cao et al. 2010). On the one hand, organic matter facilitates the adsorption of viruses by promoting hydrophobic interactions between viruses and solids and, on the other hand, it inhibits adsorption by neutralizing the positive charge of solid surfaces and by competing for adsorption sites (Kyzas et al. 2013).

Herein, our earlier work is resumed to consider the effect of the evolution of other factors along the flow paths. Let us assume *n*_1_ factors, which have an externally determined approximate common value for the whole piping system (wastewater temperature might be such a factor). In addition, let us assume *n*_2_ factors that are inert; i.e., they do not evolve along the flow path, but obey mixing rules immediately as they enter a pipe from another pipe (e.g., non adsorbed molecular or ionic species). Finally, let us assume n_3_ factors, which get adsorbed onto the solids all the way along with the flow (part of the organic matter might be such a factor). These latter type 3 factors may affect VP adsorption either exclusively in their adsorption state or in both adsorption and dissolved states. The vector of the first type of factors is denoted as **x** and it does not require governing equations since it takes externally determined values. The vector of the second type of factors is denoted as **y** and the governing equation for level 1 pipes is

17 $$ \frac{d{S}_j{V}_j{\boldsymbol{y}}_j}{d{z}_j}={\boldsymbol{Y}}_{in,j}\left({z}_j\right) $$

where ***Y***_in_ is the corresponding vector of the factor quantities entering the flow path per unit length. The situation is by far more complex for the third type of factor. Their bulk concentration is denoted by vector ***λ*** whereas their load is adsorbed onto solids by vector ***ψ*** (adsorbent quantity per mass of suspended solids). This brings about many difficulties to the problem at hand. An adsorption dynamic model similar to those for VP must be solved for each factor of type 3 in order to get its bulk and adsorbed state evolution along the flow paths. However, this implies that the bivariate particle distribution must be replaced by a multivariate one, i.e., *f(m,q,****ψ****).* Any other method of approximation for the solution of such a multivariate problem apart from the monodisperse moments method is computationally intractable. The only realistic future refinement is a better description of the distribution with respect to m only, using a higher-order moments method. The function *G* in the most general case has the dependence *G(q,C,m,****x****,****y****,****λ****,****ψ****).* There are also similar functions for each one of these factors adsorbed onto solids. These functions are represented in vectorial form as ***G***_*f*_*(q,C,m,****x****,****y****,****λ****,****ψ****)*. After application of the monodisperse moments method to the multivariate population balance, the governing equations for the type 3 factors in the level 1 flow path of index *j* become:

18 $$ \frac{d{S}_j{V}_j{\boldsymbol{M}}_{\psi, j}\left({z}_j\right)}{d{z}_j}={S}_j{\boldsymbol{G}}_f\left({q}_{ave},{C}_j,{m}_{ave},\boldsymbol{x},{\boldsymbol{y}}_j,{\boldsymbol{\lambda}}_j,{\boldsymbol{\psi}}_{ave}\right){M}_{0,0}\left({z}_j\right) $$

19 $$ \frac{d{S}_j{V}_j{\boldsymbol{\lambda}}_j}{d{z}_j}={\boldsymbol{\varLambda}}_{in,j}\left({z}_j\right)-{S}_j{\boldsymbol{G}}_f\left({q}_{ave},{C}_j,{m}_{ave}\boldsymbol{x},{\boldsymbol{y}}_j,{\boldsymbol{\lambda}}_j,{\boldsymbol{\psi}}_{ave}\right){M}_{0,0}\left({z}_j\right) $$

where the vector ***Μ***_*ψ*_ represents the concentration of the adsorbed state of the type 3 factors, ***ψ***_*ave*_ is the vector of their average adsorbed quantities per mass of solids, and ***Λ***_*in*_ is the vector of factor type 3 quantity entering the flow path per unit length.

### Model summary

**Fig. 2:**
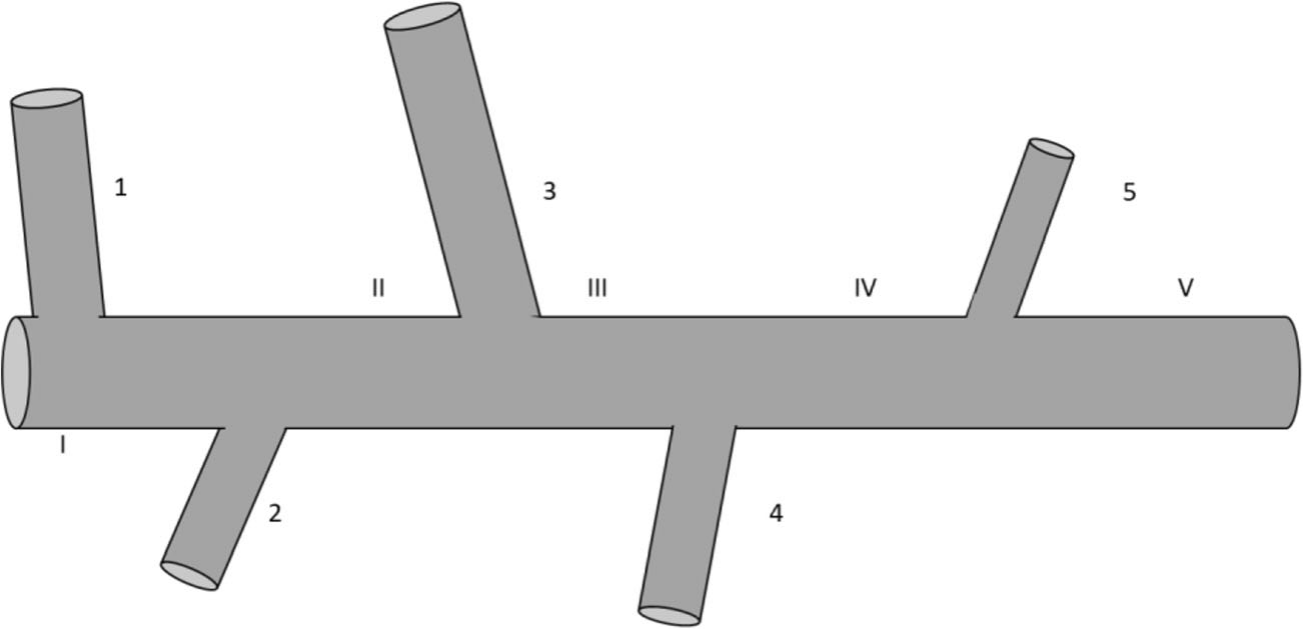
Indexing of level 1 pipes (1–5) and of main pipe sections (Ι–V) for the investigated example case

Instead of summarizing the model equations using an abstract vectorial form, it is more constructive to present them for a specific example problem involving a finite number (representative of any type) degrees of freedom. A topology is considered with *N*_L_=1 and *N*_*1*_=5 with no connectivity of the main path to pipes with a level larger than one (Fig. 2). First type factors appear only as functional dependence in G and G_f_ functions, so they are not included in the presented example. On the contrary, a second type factor and a third type factor are considered to affect the VP adsorption process. The equations of the model are as follows:

- Level 1 pipes (*j*=1,2,3,4,5)

20a $$ \frac{d{S}_j{V}_j}{d{z}_j}={F}_{in,j}\left({z}_j\right) $$

20b $$ \frac{d{S}_j{V}_j{M}_{-1,j}\left({z}_j\right)}{d{z}_j}={K}_{in,-1,j}\left({z}_j\right) $$

20c $$ \frac{d{S}_j{V}_j{M}_{0,j}\left({z}_j\right)}{d{z}_j}={K}_{in,0,j}\left({z}_j\right) $$

20d $$ \frac{d{S}_j{V}_j{C}_j}{d{z}_j}={P}_{in,j}\left({z}_j\right)-{S}_jG\left({q}_{ave},{C}_j,{m}_{ave},{y}_j,{\lambda}_j,{\psi}_{ave}\right){M}_{0,j}\left({z}_j\right) $$

20e $$ \frac{d{S}_j{V}_j{M}_{q1,j}\left({z}_j\right)}{d{z}_j}={S}_jG\left({q}_{ave},{C}_j,{m}_{ave},{y}_j,{\lambda}_j,{\psi}_{ave}\right){M}_{0,j}\left({z}_j\right) $$

20f $$ \frac{d{S}_j{V}_j{y}_j}{d{z}_j}={Y}_{in,j}\left({z}_j\right) $$

20g $$ \frac{d{S}_j{V}_j{M}_{\psi 1,j}\left({z}_j\right)}{d{z}_j}={S}_j{G}_f\left({q}_{ave},C,{m}_{ave},{y}_j,{\lambda}_j,{\psi}_{ave}\right){M}_{0,j}\left({z}_j\right) $$

20h $$ \frac{d{S}_j{V}_j{\lambda}_j}{d{z}_j}={\varLambda}_{in,j}\left({z}_j\right)-{S}_j{G}_f\left({q}_{ave},{C}_j,{m}_{ave}x,y,\lambda, {\psi}_{ave}\right){M}_{0,j}\left({z}_j\right) $$

where the vectors *y,Υ,λ,Λ,ψ,G*_*f*_ have been transformed to scalars since they include just a single element. The closure is achieved through the relations *m*_ave_=*M*_*0*_/*M*_-1_, *q*_*ave*_=*M*_*q1*_*/M*_*0*_*, ψ*_*ave*_*=M*_*ψ1*_*/Μ*_*0*_. It is preferable for variable reduction and clarity reasons to use as boundary condition a zero value for all dependent variables (concentrations and velocity) and to consider the entering material to the flow path only through the corresponding functions defined along with the flow.

#### Main flow path

The following equations must be solved between two consecutive connections of all level 1 pipes.

21a $$ \frac{d{S}_F{V}_F}{dz}=0 $$

21b $$ \frac{d{S}_F{V}_F{M}_{-1,F}(z)}{dz}=0 $$

21c $$ \frac{d{S}_F{V}_F{M}_{0,F}(z)}{dz}=0 $$

21d $$ \frac{d{S}_F{V}_F{C}_F}{dz}=-{S}_FG\left({q}_{ave},{C}_F,{m}_{ave},{y}_F,{\lambda}_F,{\psi}_{ave,F}\right){M}_{0,F}(z) $$

21f $$ \frac{d{S}_F{V}_F{M}_{q1,F}(z)}{dz}={S}_FG\left({q}_{ave},{C}_F,{m}_{ave},{y}_F,{\lambda}_F,{\psi}_{ave,F}\right){M}_{0,F}(z) $$

21g $$ \frac{d{S}_F{V}_F{y}_F}{dz}=0 $$

21h $$ \frac{d{S}_F{V}_F{M}_{\psi 1,F}(z)}{dz}={S}_F{G}_f\left({q}_{ave},{C}_F,{m}_{ave},{y}_F,{\lambda}_F,{\psi}_{ave}\right){M}_{0,F}(z) $$

21k $$ \frac{d{S}_F{V}_F{\lambda}_F}{d{z}_j}=-{S}_F{G}_f\left({q}_{av\mathrm{e}},{C}_F,{m}_{av e},{y}_F,{\lambda}_F,{\psi}_{av e}\right){M}_{0,F}(z) $$

At the five connections (*j*=1,2,3,4,5) the following balances hold:

22a $$ \varDelta \left({S}_F{V}_F\right)={\left({S}_j{V}_j\right)}_{z_j={L}_j} $$

22b $$ \varDelta \left({S}_F{V}_F{C}_F\right)={\left({S}_j{V}_j{C}_j\right)}_{z_j={L}_j} $$

22c $$ \varDelta \left({S}_F{V}_F{M}_{-1,F}\right)={\left({S}_j{V}_j{M}_{-1,j}\right)}_{z_j={L}_j} $$

22d $$ \varDelta \left({S}_F{V}_F{M}_{0,F}\right)={\left({S}_j{V}_j{M}_{0,j}\right)}_{z_j={L}_j} $$

22e $$ \varDelta \left({S}_F{V}_F{M}_{\psi 1,F}\right)={\left({S}_j{V}_j{M}_{\psi 1,j}\right)}_{z_j={L}_j} $$

22f $$ \varDelta \left({S}_F{V}_F{M}_{q1,F}\right)={\left({S}_j{V}_j{M}_{q1,j}\right)}_{z_j={L}_j} $$

22g $$ \varDelta \left({S}_F{V}_F{\lambda}_F\right)={\left({S}_j{V}_j{\lambda}_j\right)}_{z_j={L}_j} $$

22h $$ \varDelta \left({S}_F{V}_F{y}_F\right)={\left({S}_j{V}_j{y}_j\right)}_{z_j={L}_j} $$

The input variables to the above model are the functions *G* and *G*_f_, the (entering the flow paths) rate functions *F*_in_, *K*_in,-1_, *K*_*in*,0_, *Y*_in_, Λ_in_, and *P*_*in*_, the pipe cross-section area functions *S* and the pipe lengths *L*. The output of the model is the evolution of the quantities *V*, *C*, *y*, *λ*, *m*_ave_, *q*_ave_, and *ψ*_ave_ along with the flow paths.

It is recognized that the introduced notation system seems complicated at first glance but it is the most straightforward one for the requirements of the particular problem. In principle, a simplified version of the present physicochemical model could have been realized through user-defined functions in advanced software packages for multicomponent flow in wastewater piping systems. However, in that case, a completely discrete and transient approach should be employed. The discrete-continuous approach followed here leads to a huge reduction of the required computational effort. This is an essential advantage in the efficient use of the model as part of an inverse problem (i.e., estimating source shedding rates from measured concentrations in the piping system).

## Case study

Here a specific numerical example of the application of the model using realistic values of parameters is presented and analyzed. The topological structure is the one considered in the previous section having *N*_L_=1 and *N*_*1*_=5. The compositional model has an inert (type 2) factor, which facilitates VP adsorption, and an adsorbate (type 3) factor, which in the adsorbed state inhibits VP adsorption. As a first step, it is necessary to find expressions for the functions G and G_f_ which are responsible for the driving forces and kinetics of adsorption. These expressions may in principle be different among different types of viruses and their selection makes the model virus-specific. For SARS-CoV-2, adsorption expressions are not yet available. Nevertheless, for the needs of the present study, it is adequate to roughly know the size of the virus and the type of suspended solids in order to approximately define the values of the most important adsorption parameters, as explained below.

The multicomponent Langmuir-Freundlich adsorption equilibrium isotherm is employed to denote the competition for adsorption between the type 3 factor and VP (Tien 1994). In addition, a simple linear empirical relation is considered for the effect of type 2 factor on VP adsorption. It is assumed that there is no effect of the type 2 factor on the adsorption dynamics of the type 3 factor. The resulting expressions for *G*, *G*_f_ are as follows:

23a $$ G\left(q,C,m,y,\lambda, \psi \right)=\frac{15}{\frac{5r}{h_c(m)}+\frac{r^2}{D_{effc}\left(q,\psi, m\right)}}\left(\left(1+y/{y}_{ref}\right)\frac{\alpha_c{C}^{1/{n}_c}}{1+{\beta}_c{C}^{1/{n}_c}+{\beta}_{\lambda }{\lambda}^{1/{n}_{\lambda }}}-q\right) $$

23b $$ {G}_f\left(q,C,m,y,\lambda, \psi \right)=\frac{15}{\frac{5r}{h_{\lambda }(m)}+\frac{r^2}{D_{eff\lambda}\left(q,\psi, m\right)}}\left(\frac{\alpha_{\lambda }{\lambda}^{1/{n}_{\lambda }}}{1+{\beta}_c{C}^{1/{n}_c}+{\beta}_{\lambda }{\lambda}^{1/{n}_{\lambda }}}-q\right) $$

where *α*_*c*_, *α*_λ_, *β*_*c*_, *β*_*λ*_, *n*_λ_, and *n*_c_ are parameters of the multicomponent adsorption isotherm. It is convenient to express the effect of y (type 2 factor) not by another coefficient but in terms of the reference concentration *y*_*r*ef_, which is the concentration needed to double the adsorption capacity of VP (compared to *y*=0 condition).

*D*_effc_ and *D*_*e*ff λ_ are the effective diffusion coefficients of *C* (VP) and *λ* (type 3 factor) species in the solid particle of mass m having a load of *q* (of *C* component) and *ψ* (of λ component). The intraparticle structure is assumed uniform here so the effective diffusivities do not depend on particle size (i.e., on *m*). Typical empirical expressions for inhibition of the adsorbed species to effective (surface) diffusion are used (Kyzas et al. 2013):

24a $$ {D}_{effc}=\frac{D_{effc o}}{1+{d}_{c c}{q}^{n_{dc c}}+{d}_{c\lambda}{\psi}^{n_{dc\lambda}}} $$

24b $$ {D}_{eff\lambda}=\frac{D_{eff\lambda o}}{1+{d}_{\lambda c}{q}^{n_{d\lambda c}}+{d}_{\lambda \lambda}{\psi}^{n_{d\lambda \lambda}}} $$

where *D*_effco_ and *D*_effλο_ are the effective diffusivities in the unloaded (“clean”) particle and the 8 different *d* and *n*_*d*_ coefficients are empirical parameters denoting the effect of particle loading on effective diffusivities.

Finally, the external mass transfer coefficients *h*_c_ and *h*_λ_ for the two species (*C* and *λ*) differ from each other since they depend on bulk species diffusivity (needed by the dimensionless numbers in Eq. (3)). The turbulent intensity for pipe flow is approximately 0.1 (Hinze 1975) and the Kolmogorov microscale is a function of turbulent energy dissipation rate, ε, for which correlations for pipe flow exist in the literature (Friedlander 1977).

In order to decrease the huge number of the model’s degrees of freedom rationally, it is fruitful to assume that the quality of effluents is uniform along each of the 5, level 1, regional pipe streams reflecting the fact that effluents are drained from uniform (similar residential status) neighborhoods. This assumption is made only to simplify the numerical example since any kind of effluents quality can be dealt with by the model. Even under the present assumption, this quality may differ among pipes from different urban regions. The flow rate of effluents varies along each pipe depending on the residential pattern accommodated by each pipe. In order to formally code the above-described pattern, a generalized probability density function PDF(z/L) describing the distribution of effluents along the normalized length of the pipes, is employed. In this respect, the entrance to the 5 regional pipe streams, level 1, from other smaller pipes across the connected neighborhoods are expressed through the following reduced set of parameters:

25a $$ {F}_{\mathrm{in}}\left(\mathrm{z}\right)={F}_o PDF\left(z/L\right)\cdotp 1/L $$

25b $$ {K}_{\mathrm{in},-1}\left(\mathrm{z}\right)={K}_{- 1}{F}_o PDF\ \left(z/L\right)\cdotp 1/L $$

25c $$ {K}_{\mathrm{in},0}\left(\mathrm{z}\right)={K}_o{F}_o PDF\ \left(z/L\right)\cdotp 1/L $$

25d $$ {P}_{\mathrm{in}}\left(\mathrm{z}\right)={C}_o{F}_o PDF\ \left(z/L\right)\cdotp 1/L $$

25e $$ {Y}_{\mathrm{in}}\left(\mathrm{z}\right)={Y}_o{F}_o PDF\ \left(z/L\right)\cdotp 1/L $$

25f $$ {\Lambda}_{\mathrm{in}}\left(\mathrm{z}\right)={\varLambda}_o{F}_o PDF\ \left(z/L\right)\cdotp 1/L $$

where *F*_o_ is the total flow rate at *z=L*, *K*_-1_ is the number concentration of particles; *K*_o_ the mass concentration of particles; and *C*_*o*_, *Y*_*o*_, *Λ*_*ο*_ are the concentrations of species *C*, *y*, and *λ* at the entering smaller streams, respectively. The above parameters and functions are different for each pipe. It is better (and easier to measure experimentally) to use the average particle size as a second variable (the first is their mass concentration) to characterize particles instead of their number concentration. The number concentration needed in the equations can be readily calculated utilizing mass concentration, average size, and particle density.

**Fig. 3:**
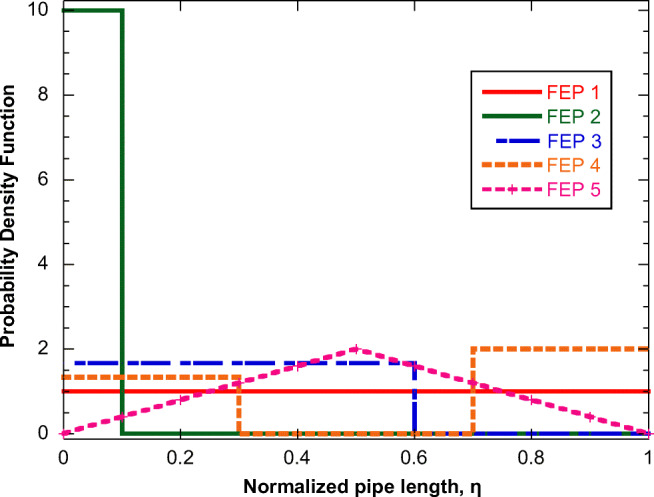
Probability density functions for the flow entrance patterns (FEP) for 1 to 5 level 1 pipes

Figure 3 presents five indicative functions PDF (distribution of effluents) versus the normalized pipe length *η=z/L* for each of the five regional pipe streams mentioned in Fig. 2. In fact, any of these five functions could be applied to any of the five regional pipe streams in Fig. 2, but for convenience, each regional pipe stream is assigned to a separate function. The list of geometric features and inlet flow conditions (total flow rate, concentrations, and average particle size) for the five regional pipe streams are shown in Table 1. The flow entrance patterns appearing in Fig. 3 are characterized by the acronym FEP and number 1 to 5 appearing in each profile in the figure.

**Table 1:** Geometric features, total flow rates, and size stream characteristics for the 5 Level 1 pipes of the investigated example case

Pipe number	Pipe diameter (m)	Pipe length (m)	Flow rate (m^3^/s)	Suspended Solids mass concentration (g/L)	Average solid equivalent diameter (mm)	VP concentration (mg/L)	Factor λ concentration (mg/L)	Factor y concentration (mg/L)
1	1.2	1800	0.5	0.7	0.7	2	1	100
2	0.8	500	0.2	1.1	0.34	4	23	200
3	1.5	2500	0.8	0.4	0.51	0.5	17	50
4	1	1600	0.4	1.8	0.29	8	13	0
5	0.8	900	0.1	1.4	0.33	12	40	0

The interdependence between the variables of the model is complicated, and since there are no experimental values for many parameters, it is useful to consider a realistic simplified case retaining major interdependencies only. So, for the present example, it is assumed that concentrations correspond to the linear regime of the isotherm (i.e., *β*_c_=*β*_λ_=0,*n*_c_=*n*_λ_=1) which means that there are available adsorption sites at every instant, a case common in nanoporous solids suspended in freshly shed wastewater. The effective diffusion coefficient for *λ* is independent of adsorbed material (*d*_λc_=*d*_λλ_=0) whereas the effective diffusion coefficient for VP depends only on *ψ* (*d*_cc_=0). In this way, the effect of *λ* factor adsorption on VP adsorption is retained and represented through the single parameter *d*_cλ_ (assuming *n*_dcλ_=1). Although the presented example, based on the parameters shown in Table 1, is useful to demonstrate the application of the model, it contains a simultaneous variation of operational variables and so it is not possible to understand the effect of each one of them separately. For this purpose, a sensitivity analysis focusing on the effect of individual variables precedes the study of the example. The sensitivity analysis defines a base case and presents the effect of a single variable variation on the evolution of VP concentration along the pipe. In particular, the effects of particle size, particle mass concentration, factor λ concentration, and flow entry pattern are examined.

The system of differential equations (20) must be solved numerically. It must be first transformed to a form directly amenable by the corresponding numerical solvers (i.e., the derivatives must be expressed with respect to dependent variables). This is done using the following derivative expansion (where X can be any one of the dependent variables):

26 $$ \frac{dSV X}{dz}= SV\frac{dX}{dz}+X\frac{dSV}{dz}= SV\frac{dX}{dz}+X{F}_{in} $$

where Eq. (20a) has been employed as an example. Accordingly, the above substitution is made to all Eqs. (20) and (21) transforming them to a regular system of ordinary differential equations (ODEs). The continuous FEP may lead to (integrable) singularities in the system equations. This means that a constant discretization step technique fails to integrate the system of ODEs. The problem is overcome by using an explicit Runge-Kutta integrator, which automatically adjusts the discretization step to a specified accuracy is reached (Press et al. 1992). For the present case, up to 400 steps have been necessary to perform the integration accurately.

**Fig. 4:**
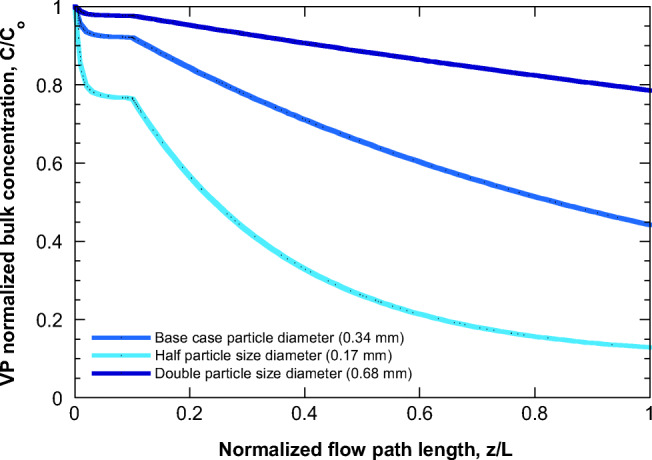
Evolution of VP normalized bulk concentration along the flow in level 1—FEP 2 pipe considering different suspended solids’ particle diameters

**Fig. 5:**
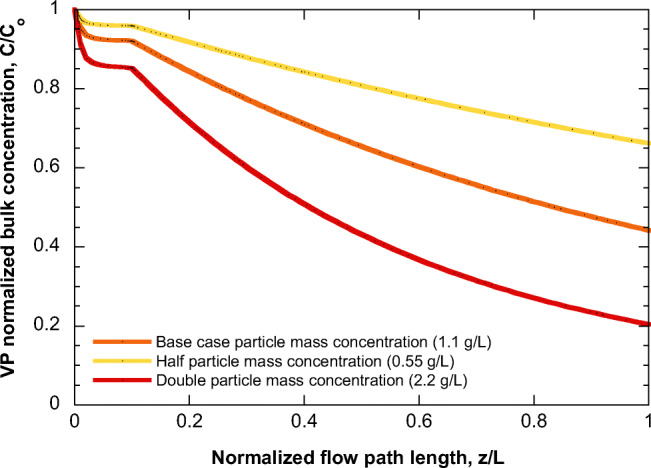
Evolution of VP normalized bulk concentration along the flow in level 1—FEP 2 pipe (base case), considering different particle mass concentrations

The base case refers to FEP 2 (flow entrance only at the first 10% of pipe length) and zero *λ* concentration. The corresponding evolution along the pipe of the normalized VP concentration is shown in Fig. 4. The flow rate close to *z*=0 is very small leading to a very large residence time. This part of the flow has enough time for adsorption, (sharp drop of C/C_o_); however, in parallel new VP material enters the flow. The combination of the above phenomena quickly leads to the establishment of a steady-state VP concentration (almost a plateau in this case) along the flow. However, at the location where the entrance flow stops (*z/L=*0.1), this steady-state is lost and adsorption dominates reducing further the bulk concentration of VP. Apparently, the effect of increasing suspended solids’ particle size is to decrease adsorption. The reason is that as the particle size increases, the solids mass refers to a smaller number of particles with a larger path for internal diffusion for VP to adsorb. The effect of suspended solids mass concentration (using the same base case as in Fig. 4) is presented in Fig. 5. Larger particle mass (at constant particle size) leads to increased adsorption and reduction of bulk VP concentration.

**Fig. 6:**
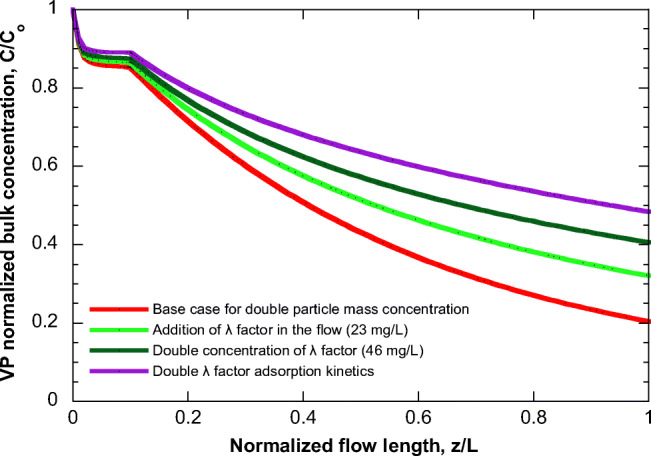
Evolution of VP normalized bulk concentration along the flow in pipe under various scenarios of *λ* factor in base case (level 1—FEP 2) considering double particle mass concentration

**Fig. 7:**
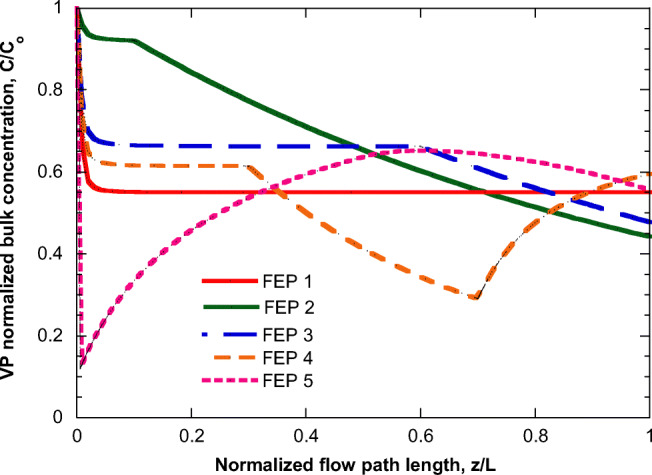
Evolution of VP normalized bulk concentration along the flow in pipe corresponding to the entrance flow PDFs appearing in Fig. 3. Base case is the same as in Figs. 4 and 5

The effect of the λ factor adsorption (happening in parallel to VP adsorption) is examined next, and the results are presented in Fig. 6. Here, as base case is used the one corresponding to curve for double particle mass concentration of Fig. 5 (high particle mass concentration) for clarity of presentation (clearer effect of *λ*). It is assumed that VP and *λ* have the same adsorption equilibrium and kinetic parameters (i.e., α_c_=α_λ_, *D*_effco_=*D*_effλο_). Figure 6 shows the effect of introducing a concentration of *λ* in the flow (curve in light green) whereas this concentration is doubled for the green curve. The purple curve holds true for the conditions of the light green curve, except for doubling the *λ* adsorption kinetics concerning VP adsorption kinetics (*D*_effλo_=2*D*_effcο_). Seemingly, the bulk VP concentration increases as the concentration of factor *λ* in the flow and its adsorption kinetics increase. Finally, the effect of FEP is presented in Fig. 7 (base case is the same as in Figs. 4 and 5). It is clear that the flow pattern radically affects the profile of the bulk VP concentration along the flow. In some cases, the addition of a new VP exceeds the VP loss by adsorption leading even to a concentration increase along the flow (e.g., FEP 4 and 5). Interestingly, for the examined cases, the final values of C/Co lie in a relatively narrow range, from 0.45 to 0.6, despite the huge differences between their profiles along the flow.

**Fig. 8:**
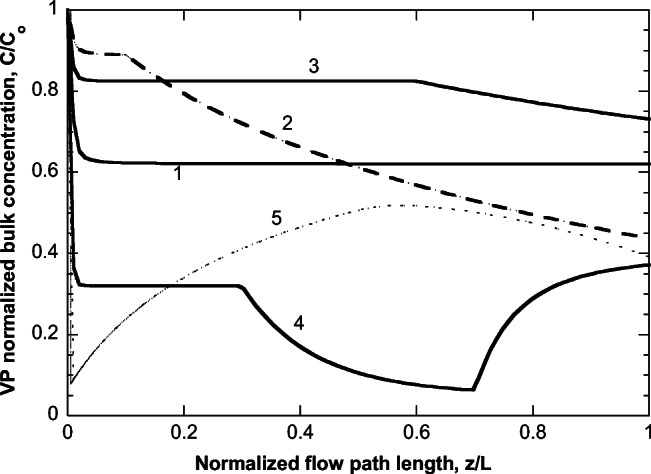
Evolution of VP normalized bulk concentration along the flow for the five level 1 pipes of the investigated example case. Pipe number is shown in the figure

**Fig. 9:**
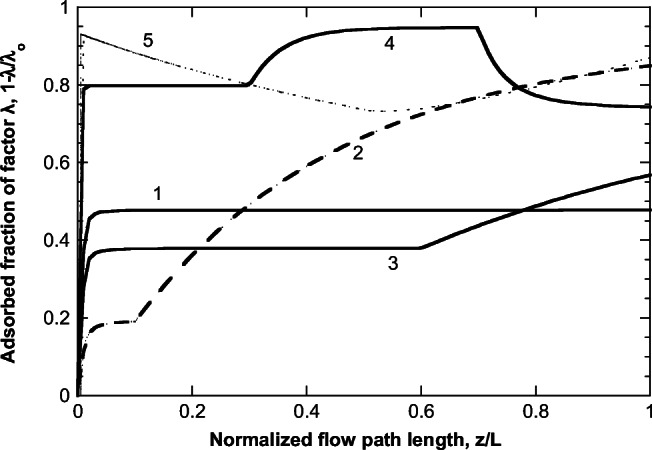
Evolution of adsorbed fraction of factor *λ* along the flow for the five level 1 pipes of the investigated example case. Pipe number is shown in the figure

The only nonzero parameters of the model used for the example treated here appear in Table 2. The effective diffusivity values assumed are representative for entities of size order of 100 nm (Deen 2012). The normalized VP bulk concentration evolution along the flow for the 5 level 1 pipes is shown in Fig. 8. The shapes of the profiles are dominated by the employed FEP patterns but the specific values at each profile are affected also by the other variables of the model that differ among pipes. The corresponding evolution of the fraction of factor *λ* concentration being in the adsorbed state appears in Fig. 9. This fraction is proportional to load ψ, which affects the diffusion of the VP into the particles and so it mirrors the profiles in Fig. 8.

**Table 2:** Parameter values considered in calculations for the investigated case.

*D*_effco_	1.34·10^-13^ m^2^/s
*D*_effλο_	2·10^-13^ m^2^/s
*α*_c_	10 l/g
*α*_λ_	10 l/g
*y*_ref_	200 mg/L
*d*_cλ_	70

**Fig. 10:**
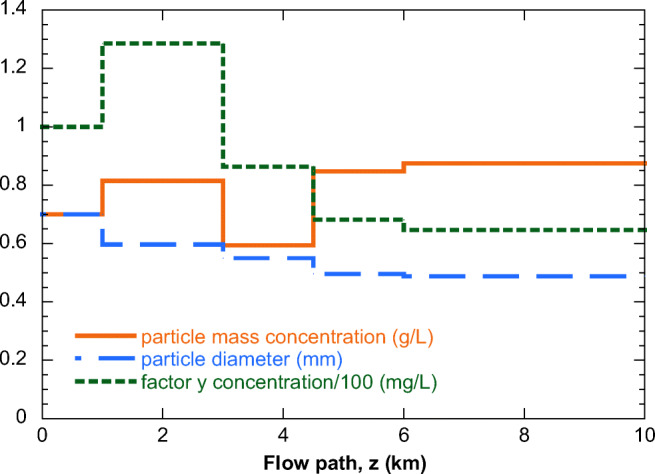
Evolution of some variables along the flow in the main pipe

We can now proceed with the study of the main pipe stream. The outlet results from the integration of the level 1 pipe models are combined to Eq. (22), expressing the balances at their connections to the main pipe. The geometric features of the main pipe sections (Fig. 2), as separated by the connections of the entering regional pipes, appear in Table 3. The equation system (21) is integrated numerically employing the balances (22) to show the evolution of variables along the flow. The profile of the suspended solids particle mass concentration, average particle size, and factor y concentration appears in Fig. 10. These quantities are constant along each pipe section since they are not affected by adsorption and there is no other mechanism to modify them. Only mixing with the entering side streams makes them vary.

**Table 3:** Geometric features of main pipe sections of the investigated example case

Section	Diameter (*m*)	Length (*m*)
I	1.6	1000
II	1.8	2000
III	2	1500
IV	2	1500
V	2	4000

**Fig. 11:**
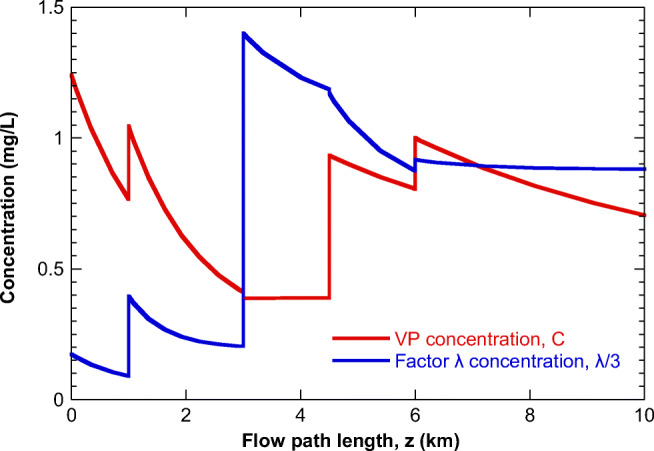
Evolution of the VP concentration (*C*) and factor *λ* concentration (*λ*) along the flow in the main pipe

The evolution of the VP and factor *λ* bulk concentrations is presented in Fig. 11. Mixing at the connections with entering regional pipes is followed by adsorption dynamics along the sections. The balance between the bulk and adsorbed VP concentrations and the properties of the solid particles at the connection between sections II and III of the main path and pipe 3 leads to desorption along section III (however, its rate is too small to become evident in the figure). In the same manner, the *λ* factor concentration remains almost constant after the last connection. Such a behavior does not necessarily mean the absence of adsorption (due to equilibrium conditions). It is probable that there is desorption from some particles and adsorption from others leading to a constant *λ* bulk concentration. In the absence of adsorption, the main pipe outlet (z=10 km) concentration of VP would be 3.3 mg/L, underlining the essential effect of adsorption on the VP concentration that can be measured at samples collected at the outlet. The large distance between the last connection and the main pipe outlet ensures that conditions at the outlet are close to equilibrium. However, a dynamic model is still necessary if intermediate measuring points are to be operated at different locations across a sewage piping network to assess the VP shedding rate at different city regions by formulating the corresponding inverse problem. Such a procedure relies greatly on the derivation of reliable parameters for the model. It is stressed that here an integrated framework is presented combining a detailed flow and physicochemical description with reasonable computational effort. Starting from a quite general situation, different simplification possibilities, in accordance with the underlying conditions, have been integrated into the present framework.

The model is a generalized framework containing a complete hierarchy of species and an increasing number of adsorption kinetic/equilibrium parameters. So there is not a specified optimum set of measurements since the number of parameters for calibration can be adjusted depending on the number of available measurements. The model has been already applied and validated (Petala et al. 2021) in the simplest possible case of an equilibrium limit (long time approximation) where measurements of virus parts concentration, flow rate, suspended solids concentration, solids particle size along with several other physicochemical parameters (corresponding to the generalized factors of the model) are available only at the outlet of the sewerage piping network. That early version of the model requires knowledge of multicomponent adsorption isotherms obtained under realistic conditions regarding competing species. The present model is an extension of the above limiting case to exploit similar measurements at several locations across the piping network where the adsorption/desorption equilibrium has not yet been established. So, further to adsorption equilibrium parameters, one should be aware of adsorption kinetic parameters and of turbulent intensity along the flow path. Assuming that one can trust existing correlations in turbulence literature, the critical step is to determine kinetic constants. All in all, the necessary step to calibrate the new model is by performing independent adsorption experiments from short to long times to determine kinetic and equilibrium adsorption parameters in a realistic matrix of different values of parameters such as virus concentration, concentration of competing species, solid particles concentration, solid particles size, dissolved oxygen concentration, and dissolved organic carbon. Although the parameters of wastewater such as the pH and electric conductivity may be very important in the adsorption process, they may not differ significantly from one location to another in a sewerage network so preliminary screening may reduce the number of experiments.

## Conclusions

The idea originally advanced in (Petala et al. 2021) of setting up an inverse problem based on the relation between the shedding rate of SARS-CoV-2 to a sewerage system and the measured concentration of virus at different locations along the pipe network is further developed in the present work. The focus here is on the fundamental aspects of the approach and specifically on the direct problem (i.e., calculation of virus concentration for a given shedding rate). This work presents the mathematical formulation (along with the necessary assumptions) for the development of a general model based on the adsorption dynamics of virus parts on solid particles suspended in wastewater, for an arbitrary topology of a sewage system. The topological complexity is reduced by introducing a hierarchical discrete-continuous structural description system. A proper parameterization of the problem is introduced, and the model is formulated as a large system of partial differential equations. The moments’ method is applied to transform the partial differential to ordinary differential equations rendering the reduced model suitable for extensive numerical calculations which are necessary for solving the inverse problem. Results of sensitivity analysis on the model are presented to show the effect of individual parameters on the results. Finally, a parametric case study for a typical system topology is performed showing the importance of adsorption on the spatial evolution of the virus concentration. It is a clear outcome of this work that relating virus concentration to shedding rate is a necessary, yet not trivial, procedure that can be performed only through models like the present one that offers a good balance between the degree of sophistication and computational effort.

## Data Availability

The datasets used and/or analyzed during the current study are available from the corresponding author on reasonable request.
